# Near-infrared induced phase-shifted ICG/Fe_3_O_4_ loaded PLGA nanoparticles for photothermal tumor ablation

**DOI:** 10.1038/s41598-017-06122-1

**Published:** 2017-07-14

**Authors:** Chengcheng Niu, Yan Xu, Senbo An, Ming Zhang, Yihe Hu, Long Wang, Qinghai Peng

**Affiliations:** 1 Department of Ultrasound Diagnosis, The Second Xiangya Hospital, Central South University, Changsha, Hunan 410011 China; 2 Department of Orthopedics, Xiangya Hospital, Central South University, Changsha, Hunan 410008 China

## Abstract

Near-infrared (NIR) laser-induced photothermal therapy (PTT) uses a photothermal agent to convert optical energy into thermal energy and has great potential as an effective local, minimally invasive treatment modality for killing cancer cells. To improve the efficacy of PTT, we developed poly(lactide-co-glycolide) (PLGA) nanoparticles (NPs) encapsulating superparamagnetic iron oxide (Fe_3_O_4_), indocyanine green (ICG), and perfluoropentane (PFP) as synergistic agents for NIR laser-induced PTT. We fabricated a novel type of phase-shifting fluorescent magnetic NPs, Fe_3_O_4_/ICG@PLGA/PFP NPs, that effectively produce heat in response to NIR laser irradiation for an enhanced thermal ablation effect and a phase-shift thermoelastic expansion effect, and thus, can be used as a photothermal agent. After *in vitro* treatment of MCF-7 breast cancer cells with Fe_3_O_4_/ICG@PLGA/PFP NPs and NIR laser irradiation, histology and electron microscopy confirmed severe damage to the cells and the formation of many microbubbles with iron particles at the edge or outside of the microbubbles. *In vivo* experiments in mice with MCF-7 tumors demonstrated that Fe_3_O_4_/ICG@PLGA/PFP NPs could achieve tumor ablation upon NIR laser irradiation with minimal toxicity to non-irradiated tissues. Together, our results indicate that Fe_3_O_4_/ICG@PLGA/PFP NPs can be used as effective nanotheranostic agents for tumor ablation.

## Introduction

Photothermal therapy (PTT) employs a near-infrared (NIR) laser and photo-absorbing agents to generate heat from light energy to “burn” cancer cells^1, 2^. Compared with traditional treatments, such as chemotherapy, radiotherapy and surgery, PTT is a less invasive and highly effective therapeutic approach applied for local treatment of tumors. With high intensity focused ultrasound (HIFU) ablation, the high acoustic power may cause severe side effects in normal tissue in the ultrasound propagation channel because a high temperature can be generated in any tissue, not only in the presence of the nanoparticles. In contrast, for PTT to generate a high temperature in tissues, the photo-absorbing nanoparticles must be present, and thus, the effect more accurately damages the targeted legions^3, 4^. Several nanomaterials with excellent NIR light absorption properties have been developed as PTT agents, including gold nanomaterials^5–12^, copper nanomaterials^13–18^, carbon nanomaterials^19–23^, and NIR dyes^24–27^. However, the potential toxicity of non-degradable or slowly degradable nanoparticles (NPs), especially carbon nanotubes or gold nanoparticles, will inevitably limit their clinic applications^28–30^. Thus, there is a significant need to develop a biologically safe and biodegradable photoabsorber for the NIR laser-induced PTT.

Indocyanine green (ICG), as the only NIR agent approved by the U.S. Food and Drug Administration (FDA) to date, has been widely used for human clinical NIR fluorescence (NIRF) imaging and diagnosis^31, 32^. Several challenges exist with regard to the use of ICG in PTT applications though, such as its instability in aqueous solution, light- and temperature-dependent properties, and rapid blood clearance^33, 34^. To overcome these limitations, researchers have developed polymeric NP delivery systems for encapsulating ICG^35, 36^. Several studies have shown that encapsulation of ICG in NPs can enhance the stability of ICG for *in vivo* NIRF imaging and increase its accumulation in tumors through an “enhanced permeability and retention effect” (EPR effect)^37–40^. Therefore, the efficacy of PTT can be enhanced by the use of NP-encapsulated ICG.

Magnetic iron oxide (Fe_3_O_4_) NPs have received great attention for their low toxicity, strong effects on T_2_ and ${{\rm{T}}}_{2}^{\ast }$ relaxation for magnetic resonance imaging (MRI), and excellent photo-absorbing ability^1, 2, 41, 42^. However, individual magnetic nanoparticles are susceptible to opsonization in the bloodstream, which will lead to uptake by the reticuloendothelial system (RES) and quick clearance from the blood, resulting in limited distribution in target tissue^43, 44^, and thereby hindering the imaging or PTT efficacy. Many polymers systems have been investigated for use in decorating the magnetic NPs to address this issue^45, 46^. Poly (lactide-co-glycolide) (PLGA) is one of the most widely used polymers for its biodegradability and biocompatibility as well as approval by the FDA. Previously, we developed PLGA microbubbles loaded with doxorubicin and Fe_3_O_4_ NPs and demonstrated their suitability for dual MR/ultrasound (MR/US) imaging of sentinel lymph nodes and their anti-tumor efficacy for lymph node metastasis^47^.

Recently, many studies have focused on liquid perfluorocarbon (PFC) droplets, which can be vaporized into gas bubbles via active US sonication or laser irradiation^48, 49^. This property of vaporization has been effectively employed for US imaging^50^, cancer therapy via vessel occlusion^51–53^, targeted drug delivery^54–56^, and thermal ablation of tumor cells^57, 58^. However, this technology has yet to be combined with a dual NIR light-absorbing agent along with encapsulation in polymeric NPs toward the development of an effective PTT approach.

**Figure 1:**
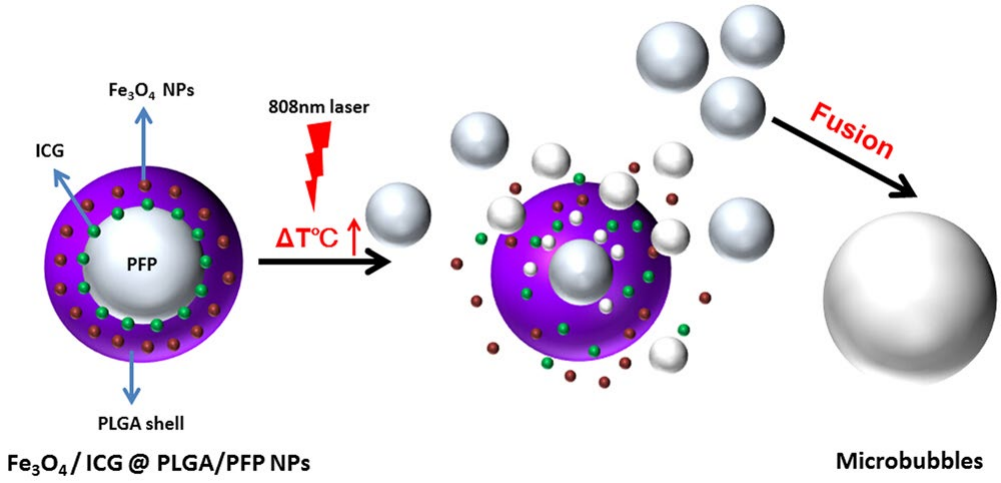
Schematic illustration of the structure of Fe_3_O_4_/ICG@PLGA/PFP NPs and the progress of the generation of mircobubbles from within the NPs via NIR-induced vaporization of PFP.

Here, we report the development of novel pefluoropentane (PFP)-based PLGA NPs loaded with Fe_3_O_4_ NPs and ICG (Fe_3_O_4_/ICG@PLGA/PFP NPs) for photothermal tumor ablation (Fig. 1). Both Fe_3_O_4_ NPs and ICG contribute to the ability of this NIR light-absorbing agent to efficiently convert absorbed light into heat. The liquid PFP core with a lower boiling point is easily converted into gas upon heating to physiological temperatures, and this thermoelastic expansion results in tissue extrusion deformation to enhance the thermal ablation effect in the local tumor area. Our results show that these dual NIR light-absorbing PFC-based polymeric NPs can be used as effective nanotheranostic agents in anti-tumor treatments.

## Materials and Methods

### Materials

ICG, poly vinyl alcohol (PVA, Mw = 30,000–70,000), and PLGA (lactide: glycolide = 50:50, Mw = 10,000) were obtained from Sigma-Aldrich (USA). Fe_3_O_4_ NPs (diameter = 10 nm) treated with oleic acid were purchased from Ocean Nano Tech Inc. (USA). PFP was purchased from Alfa Aesar (UK). De-ionized (DI) water was purified using a Milli-Q Gradient System. Other regents of analytical grade were used without further purification. All experiments involving use of animals were performed in accordance with the relevant guidelines and regulations, approved by the Ethics Committee at the second Xiangya Hospital of Central South University in China.

### Preparation of Fe_3_O_4_/ICG@PLGA/PFP NPs

The Fe_3_O_4_/ICG@PLGA/PFP NPs were fabricated using a slightly modified double emulsion method^47^. Briefly, 500 mg PLGA and 2 mL Fe_3_O_4_ NPs suspension (31 mg Fe/mL) were added to 10 mL of chloroform and stirred well, and then 2 mL ICG solution (1.25 mg/mL) and 4 mL liquid PFP were added and emulsified for 1 min in an ice bath using an ultrasonic processor. Then 50 mL cold PVA solution (5% w/v) was poured into the emulsion, which was homogenized for 5 min at 9500 rpm in an ice bath using a homogenizer. Subsequently, 100 mL DI water was added to the mixture and mixed for 2 h. Finally, the NPs were washed with DI water three times and kept at 4 °C until characterization. The same procedure was used to prepare Fe_3_O_4_/ICG@PLGA NPs without PFP, Fe_3_O_4_ @PLGA/PFP NPs without ICG, and ICG@PLGA/PFP NPs without Fe3O4 as controls.

### Characterization of Fe_3_O_4_/ICG@PLGA/PFP NPs

The morphology of Fe_3_O_4_/ICG@PLGA/PFP NPs was observed by scanning electron microscopy (SEM, JEOL-7800F). The structure and the presence of Fe_3_O_4_ NPs in the NP shell were confirmed by transmission electron microscopy (TEM, Hitachi H-7600). The size distribution of NPs was analyzed using a Nano ZS dynamic light scattering analyzer (Malvern Instruments, UK). The amount of elemental Fe encapsulated in the NPs was determined by atomic absorption spectrometry. The UV-Vis-NIR absorption spectra of free ICG, Fe_3_O_4_ NPs and Fe_3_O_4_/ICG@PLGA/PFP NPs (0.2 mL of each sample, equivalent ICG concentration of 5 μg/mL and equivalent Fe concentration of 113 μg/mL) were obtained using a steady state spectrophotometer (QuantaMaster^TM^ 40, USA) at room temperature to ensure high absorption in the NIR region and to monitor the ICG content. The encapsulation efficiency of ICG in Fe_3_O_4_/ICG@PLGA/PFP NPs was calculated as the weight ratio of encapsulated ICG to the total added ICG amount^43^. The amount of encapsulated ICG was calculated as the difference between the total added ICG amount and the amount recovered in washed supernatants during preparation.

### Temperature elevation induced by NIR laser irradiation

The temperature profile of Fe_3_O_4_/ICG@PLGA/PFP NPs was monitored under laser irradiation using an infrared thermometer (Fluke 62 MAX, USA). Aqueous suspensions of Fe_3_O_4_/ICG@PLGA/PFP NPs, mixture of ICG and Fe_3_O_4_ NPs, free ICG, Fe_3_O_4_ NPs, and phosphate-buffered saline (PBS; as the negative control) in wells of a 96-well plate (0.2 mL/well) were irradiated by a NIR 808 nm laser at 1.0 W/cm^2^ for 10 min (T808F2W, Xi’an Minghui Optoelectronic Technology, China). The amount of ICG was equivalent in the free ICG, mixture of ICG and Fe_3_O_4_ NPs, and Fe_3_O_4_/ICG@PLGA/PFP NPs samples (5 μg/mL ICG)^1, 55^, and the amount of Fe_3_O_4_ was equivalent in the Fe_3_O_4_ NPs, mixture of ICG and Fe_3_O_4_ NPs, and Fe_3_O_4_/ICG@PLGA/PFP NPs samples (113 μg/mL Fe). The temperatures of the solutions were measured at 30-s intervals.

### Photostability of Fe_3_O_4_/ICG@PLGA/PFP NPs

The photostability of Fe_3_O_4_/ICG@PLGA/PFP NPs was tested out under 808 nm NIR laser ON/OFF cycle irradiation. For comparison with free ICG, Fe_3_O_4_/ICG@PLGA/PFP NPs, CG@PLGA/PFP NPs, and free ICG were irradiated for 3 min (Laser ON), followed by naturally cooling for 10 min (Laser OFF). This ON/OFF cycle was repeated four times, and the temperatures of the solutions were measured at 30-s intervals.

### *In vitro* cell experiments

The cytotoxicity of Fe_3_O_4_/ICG@PLGA/PFP NPs was evaluated using cell counting kit (CCK-8) assays with the human breast cancer MCF-7 cell line. MCF-7 cells (10^4^ cells/well) were incubated in 96-well plates at 37 °C for 24 h. Different dosages (0.2, 0.4, 0.6 and 0.8 mg/mL, 0.1 mL/well) of Fe3O4/ICG@PLGA/PFP NPs suspension were added for incubation for 6 h. Medium without Fe_3_O_4_/ICG@PLGA/PFP NPs was used as a control. The cells in each experimental well were exposed to 808 nm laser irradiation (1 W/cm^2^) for 5 min, and control samples were exposed to laser irradiation. Cell viability was determined using the CCK assay.

For Hoechst 33342/propidium iodide (PI) staining, MCF-7 cells (10^5^ cells/well) were incubated in 6-well plates at 37 °C with 0.5 mg/mL Fe_3_O_4_/ICG@PLGA/PFP NPs and then irradiated by the NIR laser (808 nm, 1 W/cm^2^) for 5 min. The cells were stained with a mixed solution of Hoechst 33342 and PI at 4 °C for 30 min and examined under a fluorescence microscope (Olympus IX71, Japan) to verify the photothermal effect on the cancer cells. The excitation wavelength was set at 355 nm for Hoechst 33342 and 545 nm for PI.

### *In vivo* PTT

Female BALB/c nude mice (20 g, age: 6–8 weeks) were obtained from the Medical Experimental Animal Center of the Second Xiangya Hospital of Central South University (Changsha, China). Animals received care in accordance with the Guidance Suggestions of the Ethics Committee of the Second Xiangya Hospital of Central South University in China. To establish the animal breast tumor model, 1 × 10^6^ MCF-7 cells were administered by subcutaneous injection into the right flank of the nude mice.

When tumor volumes reached about 60 mm^3^, mice were randomly divided into four treatment groups (n = 6 per group): (I) saline; (II) Fe_3_O_4_/ICG@PLGA/PFP NPs alone without laser irradiation (NPs only); (III) laser irradiation only without Fe_3_O_4_/ICG@PLGA/PFP NPs (laser only); and (IV) Fe_3_O_4_/ICG@PLGA/PFP NPs combined with laser irradiation (NPs + laser). In groups I, II, and IV, the nude mice received an intratumor injection of 50 µL saline or Fe_3_O_4_/ICG@PLGA/PFP NPs (2 mg/mL). Two hours after injection, the tumors of mice in groups III (laser only) and IV (NPs + laser) were irradiated with the 808 nm laser at a power density of 1.0 W/cm^2^ for 5 min. The mice in groups I (saline) and II (NPs only) received no laser irradiation as controls. The temperature changes in the tumors were monitored by an infrared thermal imaging camera (Ti27, Fluke, USA). The tumor volume was calculated according to the formula: (tumor width)^2^ × (tumor length)/2. The tumor size in each mouse was recorded every 2 days for 14 days.

One day after the different treatments, tumor tissues were harvested and evaluated via hematoxylin and eosin (H&E) staining, TdT-mediated dUTP nick end labeling (TUNEL) staining, and TEM examination for assessment of therapeutic efficacy.

### Biological toxicity assessment

For assessment of the biological toxicity of the developed NPs, 10 healthy female BALB/c nude mice were intravenously injected with 0.2 mL of 2 mg/mL Fe_3_O_4_/ICG@PLGA/PFP NPs. Five additional mice were injected with saline as controls. Blood samples were collected for serum biochemistry assays at 3 and 14 days after injection of Fe_3_O_4_/ICG@PLGA/PFP NPs. After sacrifice at the final time point, the major organs including the liver, spleen, kidney, heart, lung and brain were harvested, and sections were stained with H&E.

### Statistical analysis

All data are presented as means ± standard deviation (SD). Analysis of variance was used to analyze the data. Differences were considered significant if *p* < 0.05.

## Results and Discussion

### Structural characterization of Fe_3_O_4_/ICG@PLGA/PFP NPs

**Figure 2:**
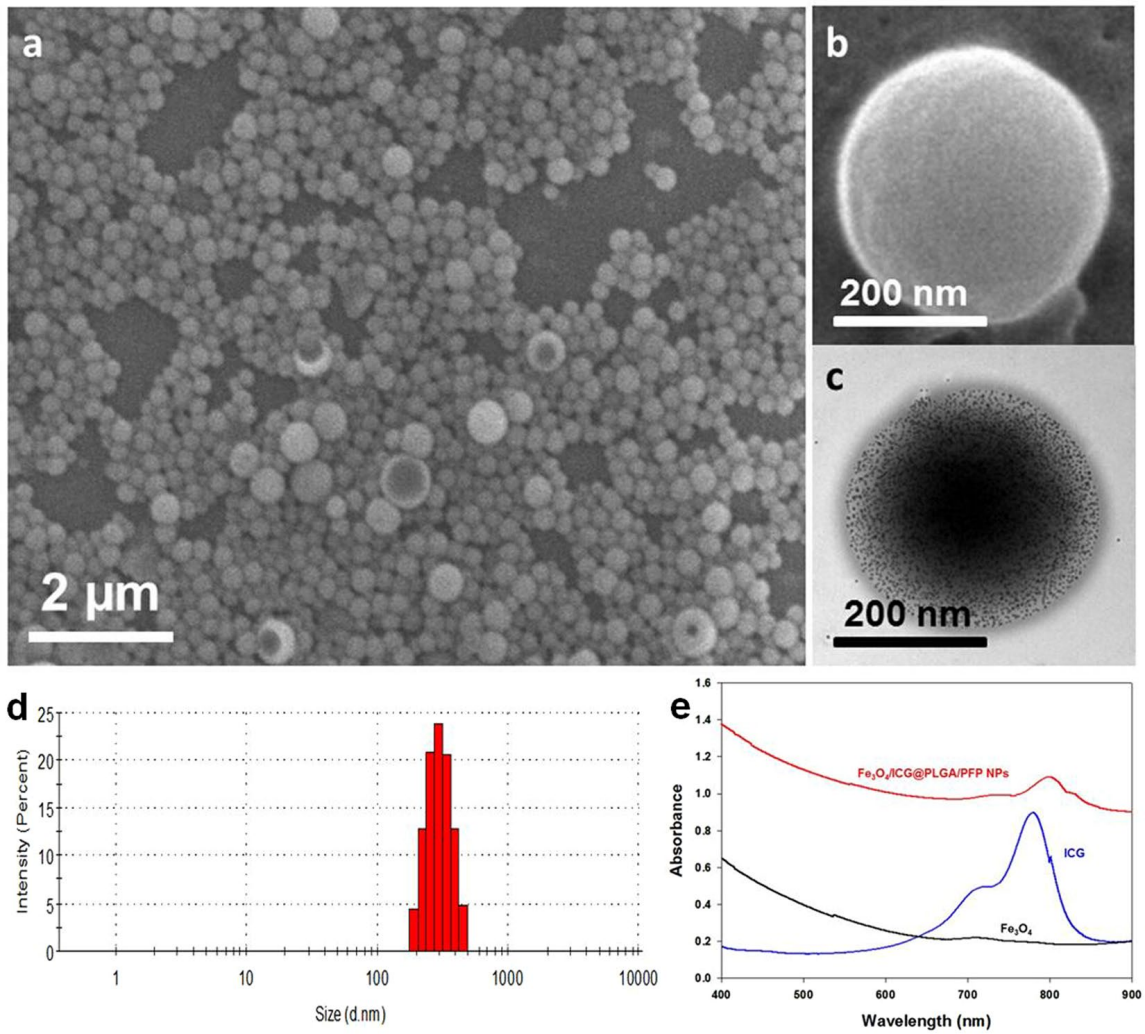
(**a**,**b**) SEM images of Fe_3_O_4_/ICG@PLGA/PFP NPs (**a**) 10000×; scale bar, 2 μm; (**b**) 70000×; scale bar, 200 nm); (**c**) TEM image of Fe_3_O_4_/ICG@PLGA/PFP NPs with a large amount of black Fe_3_O_4_ NPs embedded in the spherical shell (70000×; scale bar, 200 nm); (**d**) size distribution of Fe_3_O_4_/ICG@PLGA/PFP NPs with the average diameter of 289.6 ± 67.4 nm; (**e**) UV-Vis-NIR absorption spectra of Fe_3_O_4_/ICG@PLGA/PFP NPs, free ICG and Fe_3_O_4_ NPs.

The Fe_3_O_4_/ICG@PLGA/PFP NPs were fabricated using our previously published method with a small change in the continuous sonication time from 40 s to 1 min^47^. The morphology and structure of Fe_3_O_4_/ICG@PLGA/PFP NPs were characterized by SEM and TEM. The Fe_3_O_4_/ICG@PLGA/PFP NPs appeared well dispersed and spherical in shape with a smooth surface morphology (Fig. 2a,b), average diameter of 289.6 ± 67.4 nm, and polydispersity index of 0.028 (Fig. 2d). In TEM (Fig. 2c), scattered black spots with diameter of 10 nm were clearly observed on the shells of Fe_3_O_4_/ICG@PLGA/PFP NPs, confirming the encapsulation of Fe_3_O_4_ NPs in the Fe_3_O_4_/ICG@PLGA/PFP NPs. Furthermore, the amount of Fe_3_O_4_ NPs encapsulated in the Fe_3_O_4_/ICG@PLGA/PFP NPs was determined by atomic absorption spectrometry to be 113.55 ± 3.12 μg/mL. The encapsulation efficiency of ICG was 45.53% ± 1.61% as measured by steady state spectrophotometry. The UV-Vis-NIR absorption spectra of Fe_3_O_4_/ICG@PLGA/PFP NPs, ICG, and Fe_3_O_4_ NPs displayed strong absorption in PBS in the range of 400–900 nm (Fig. 2e). The spectrum of Fe_3_O_4_ NPs exhibited no obvious peak, whereas that of free ICG had a peak around 770~790 nm. The absorption spectrum of Fe_3_O_4_/ICG@PLGA/PFP NPs displayed a small peak around 790~810 nm with an obvious red shift, which is consistent with previously published results for ICG^33, 57–61^, confirming that ICG was successfully encapsulated in the NPs. The observed absorbance in the NIR region verified that the Fe_3_O_4_/ICG@PLGA/PFP NPs could serve as a good photo-absorbing agent for tumor PTT.

### *In vitro* PTT effect of Fe_3_O_4_/ICG@PLGA/PFP NPs

**Figure 3:**
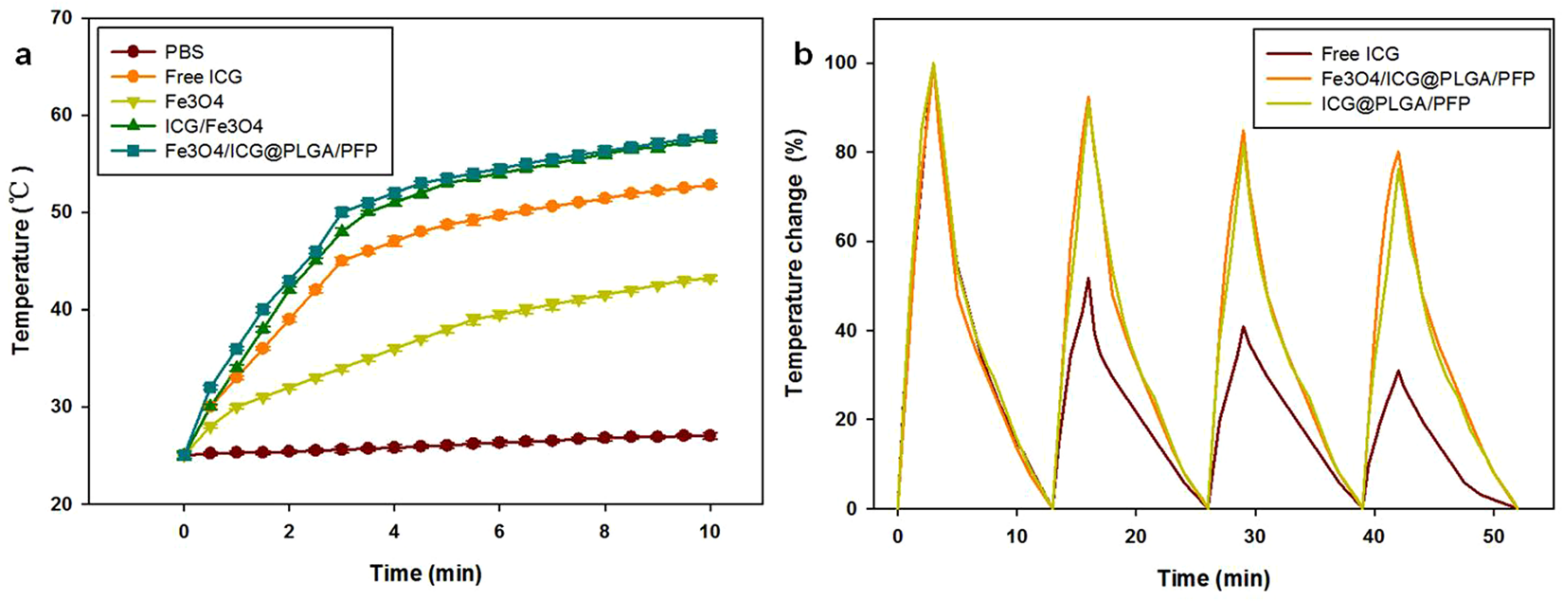
*In vitro* photothermal effects of Fe_3_O_4_/ICG@PLGA/PFP NPs. (**a**) Temperatures elevation of Fe_3_O_4_/ICG@PLGA/PFP NPs, mixture of free ICG and Fe_3_O_4_ NPs, free ICG, Fe_3_O_4_ NPs, and PBS with continuous NIR laser irradiation (808 nm, 1.0 W/cm^2^, 10 min). (**b**) Temperature changes of Fe_3_O_4_/ICG@PLGA/PFP NPs, ICG@PLGA/PFP NPs, and free ICG over four NIR laser ON/OFF cycles.

To study the effectiveness of using Fe_3_O_4_/ICG@PLGA/PFP NPs in PTT, aqueous suspensions of Fe_3_O_4_/ICG@PLGA/PFP NPs, mixture of ICG and Fe_3_O_4_ NPs, free ICG, Fe_3_O_4_ NPs, and PBS were exposed to 808 nm NIR laser irradiation with a power density of 1.0 W/cm^2^ for 10 min. The rapidly increasing temperature of Fe_3_O_4_/ICG@PLGA/PFP NPs allowed them to act as an effective photothermal nanoagent for PTT. As shown in Fig. 3a, no obvious temperature change was observed when PBS was exposed to NIR laser irradiation. In contrast, under the same concentration (0.2 mL solution with 5 μg/mL ICG or 113 μg/mL Fe) and laser irradiation conditions, the maximum temperatures achieved in the free ICG, Fe_3_O_4_ NPs, mixture of ICG and Fe_3_O_4_ NPs, and Fe_3_O_4_/ICG@PLGA/PFP NP solutions were 52.8 °C, 43.2 °C, 57.0 °C, and 57.9 °C, respectively. The higher maximum temperature observed for the Fe_3_O_4_/ICG@PLGA/PFP NPs and the mixture of ICG and Fe_3_O_4_ NPs indicates that Fe_3_O_4_/ICG@PLGA/PFP NPs and the combination of ICG and Fe_3_O_4_ NPs may be more effective than pure ICG or Fe_3_O_4_ NPs in PTT. This result indicates that both the ICG and Fe_3_O_4_ NPs entrapped in the Fe_3_O_4_/ICG@PLGA/PFP NPs contribute to the NIR-dependent temperature increase and that Fe_3_O_4_/ICG@PLGA/PFP NPs could act as efficient NIR light absorbers for PTT.

To compare the photostability of Fe_3_O_4_/ICG@PLGA/PFP NPs with that of the free ICG molecules, solutions of Fe_3_O_4_/ICG@PLGA/PFP NPs, ICG@PLGA/PFP NPs and ICG were irradiated with the NIR laser for 3 min (Laser ON), followed by natural cooling for 10 min (Laser OFF). After each of the four laser ON/OFF cycles, as shown in Fig. 3b, the temperature increases in the Fe_3_O_4_/ICG@PLGA/PFP NPs solution were 25.1 °C, 23.2 °C, 21.3 °C and 20.1 °C; the temperature increases in the ICG@PLGA/PFP NP solution were 21.1 °C, 19.2 °C, 17.3 °C, and 16.1 °C; and those for the free ICG solution were 20.3 °C, 10.5 °C, 8.3 °C, and 6.3 °C. The greater increase in the temperature of the ICG@PLGA/PFP NP solution indicated that ICG loaded in the polymeric layers NPs show greater photostability than the free ICG molecules. The temperature increases in the Fe_3_O_4_/ICG@PLGA/PFP NP solution were greater than those in the ICG@PLGA/PFP NP solution, which showed that Fe_3_O_4_ in the former NPs also contributed to the photothermal conversion efficiency. This result is consistent with the findings of a previous study by Dai *et al*.^1^ concerning lipid shell NPs.

### *In vitro* cytotoxicity of Fe_3_O_4_/ICG@PLGA/PFP NPs

**Figure 4:**
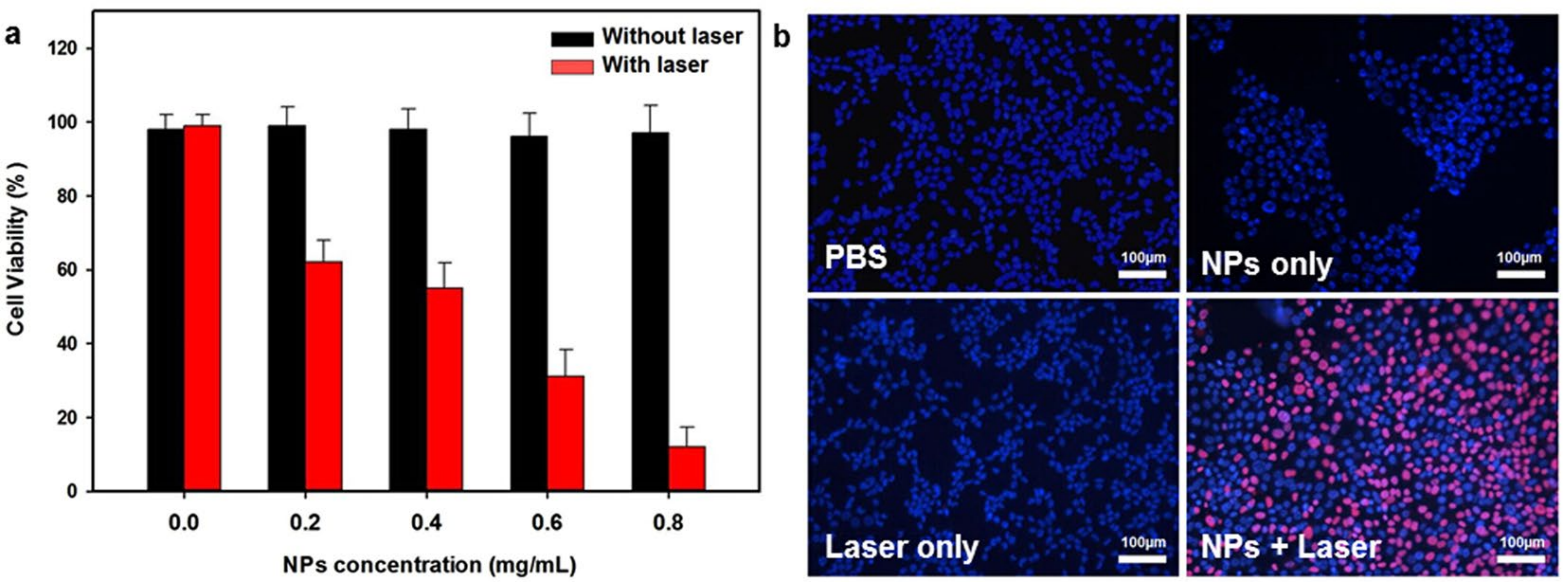
*In vitro* cytotoxicity experiments. (**a**) Relative viability of MCF-7 cells incubated with different concentrations of Fe_3_O_4_/ICG@PLGA/PFP NPs under laser irradiation (808 nm, 1 W/cm^2^, 5 min). (**b**) Fluorescence images of Hoechst 33342/PI co-stained MCF-7 cells incubated with Fe_3_O_4_/ICG@PLGA/PFP NPs (0.5 mg/mL) and exposed to the 808 nm laser irradiation (1 W/cm^2^, 5 min). Live and dead cells were stained with Hoechst 33342 (blue color) and PI (red color), respectively.

To investigate the photothermal cytotoxicity of Fe_3_O_4_/ICG@PLGA/PFP NPs, MCF-7 cells were incubated with various concentrations of Fe_3_O_4_/ICG@PLGA/PFP NPs for 6 h and then treated with or without 808 nm laser irradiation at a power density of 1.0 W/cm^2^ for 5 min before cell viability was measured via CCK-8 assay. Cells not exposed to NPs and irradiated or not served as the controls. As shown in Fig. 4a, no obvious decrease in cell viability was observed upon exposure to various concentrations of Fe_3_O_4_/ICG@PLGA/PFP NPs without laser irradiation. In contrast, enhanced cancer cell ablation was observed with increasing concentrations of Fe_3_O_4_/ICG@PLGA/PFP NPs under NIR laser irradiation.

To further confirm the photothermal effect of Fe_3_O_4_/ICG@PLGA/PFP NPs, viable and dead cells within MCF-7 cell samples were stained with Hoechst 33342 and PI, respectively. As shown in Fig. 4b, many dead cells (red fluorescent) were observed only after treatment with both Fe_3_O_4_/ICG@PLGA/PFP NPs and laser irradiation, indicating that some cells underwent photothermal destruction. In comparison, samples from the control groups showed blue staining over the entire area, suggesting that treatment with Fe_3_O_4_/ICG@PLGA/PFP NPs or laser irradiation alone did not induce cell death. These results demonstrate that the Fe_3_O_4_/ICG@PLGA/PFP NPs could effectively kill the tumor cells through PTT only upon NIR laser irradiation.

### Use of Fe_3_O_4_/ICG@PLGA/PFP NPs in PTT for tumor ablation *in vivo*

**Figure 5:**
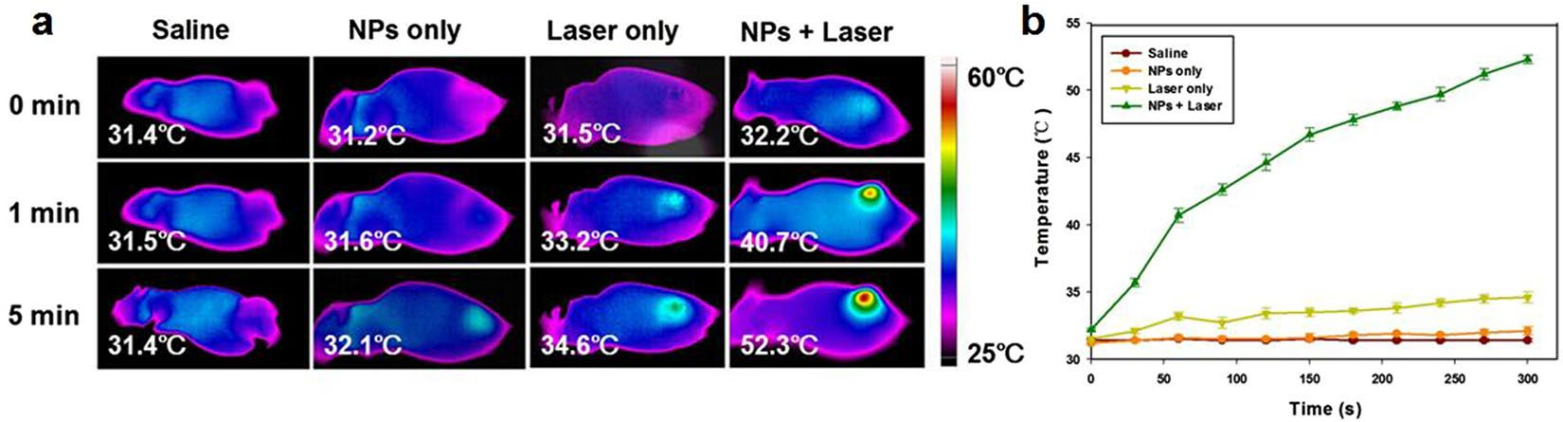
*In vivo* PTT effectiveness of Fe_3_O_4_/ICG@PLGA/PFP NPs. (**a**) IR thermal images showing the temperature changes in tumors in mice that received different treatments. (**b**) Peak temperature profiles in tumors with different treatments.

To further study the potential of Fe_3_O_4_/ICG@PLGA/PFP NPs for *in vivo* cancer PTT, mice bearing MCF-7 cell tumors were subjected to four different treatments: saline, Fe_3_O_4_/ICG@PLGA/PFP NPs only, laser irradiation only, Fe_3_O_4_/ICG@PLGA/PFP NPs and laser irradiation. As monitored by an infrared thermal camera, the temperature of tumor tissue injected with Fe_3_O_4_/ICG@PLGA/PFP NPs increased rapidly from 32.2 °C to 52.3 °C under laser irradiation. In contrast, no temperature increases were observed in tumors after treatment with saline, NPs only, or laser irradiation only (Fig. 5a and b).

**Figure 6:**
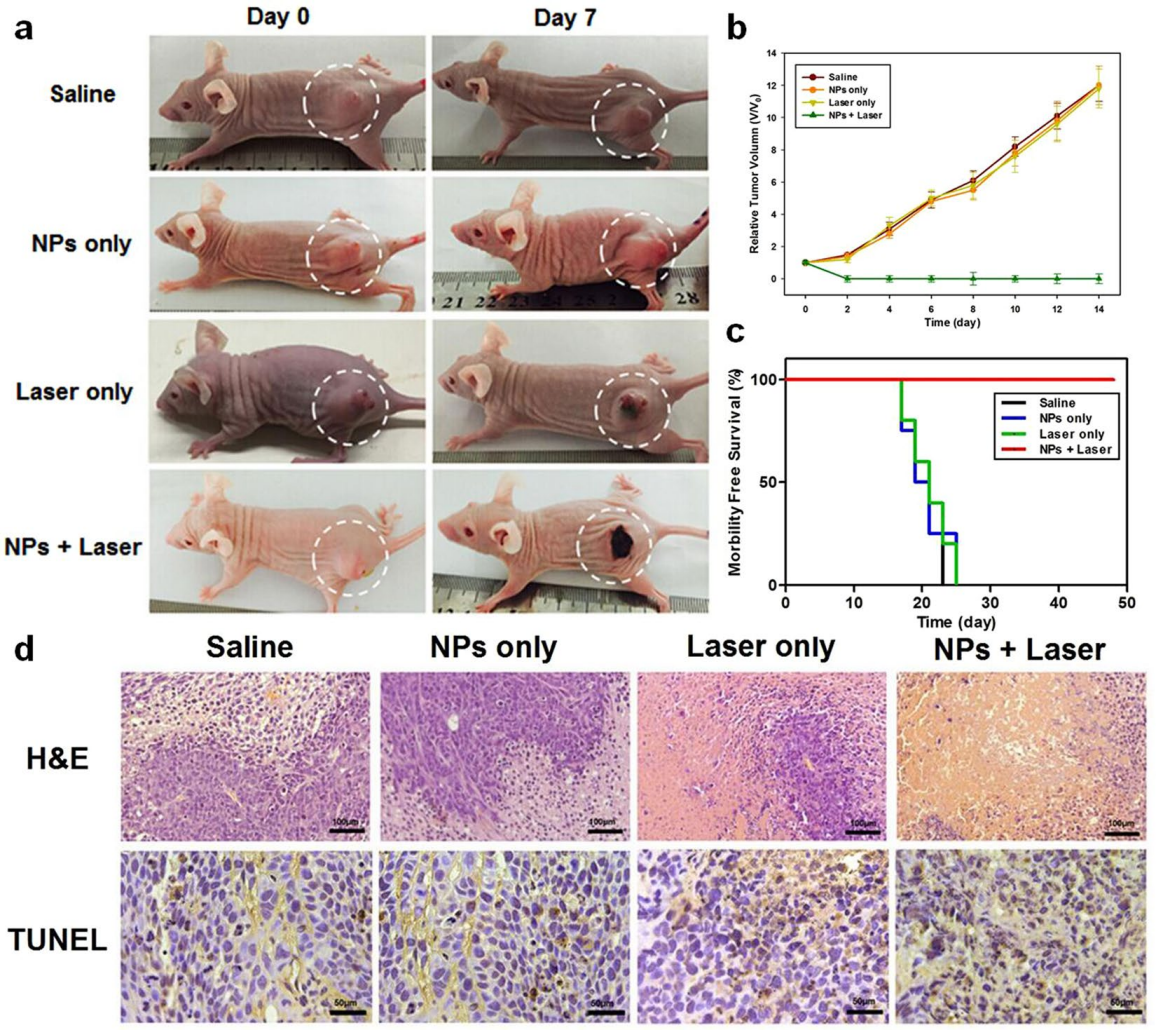
*In vivo* cancer therapy. (**a**) Representative photographs of mice from different groups at day 0 before treatment and at day 7 after treatment. (**b**) Tumor growth curves of mice after different treatments indicated. (**c**) Morbidity-free survival data after different treatments. (**d**) H&E and TUNEL staining of sections of tumors harvested 1 day after the different treatments. (H&E staining: 100×; scale bar, 100 μm; TUNEL staining: 200×; scale bar, 50 μm).

The photographs in Fig. 6a show the black scars that appeared over the sites of tumors injected with Fe_3_O_4_/ICG@PLGA/PFP NPs upon laser irradiation. In these mice, the tumor size declined significantly, and the scar tissue gradually fell off within 14 days laser irradiation. However, in the three control groups treated with saline only, NPs only, or laser irradiation only, the volumes of tumors increased by more than 12-fold from day 0 to day 14 after treatment (Fig. 6b). Moreover, mice in the three control groups showed a survival range of 17–23 days after treatment, whereas all mice treated with Fe_3_O_4_/ICG@PLGA/PFP NP injection and laser irradiation remained alive at 48 days after treatment with complete tumor ablation achieved by PTT (Fig. 6c). These results demonstrate that Fe_3_O_4_/ICG@PLGA/PFP NPs could effectively inhibit tumor growth via their photothermal efficacy.

**Figure 7:**
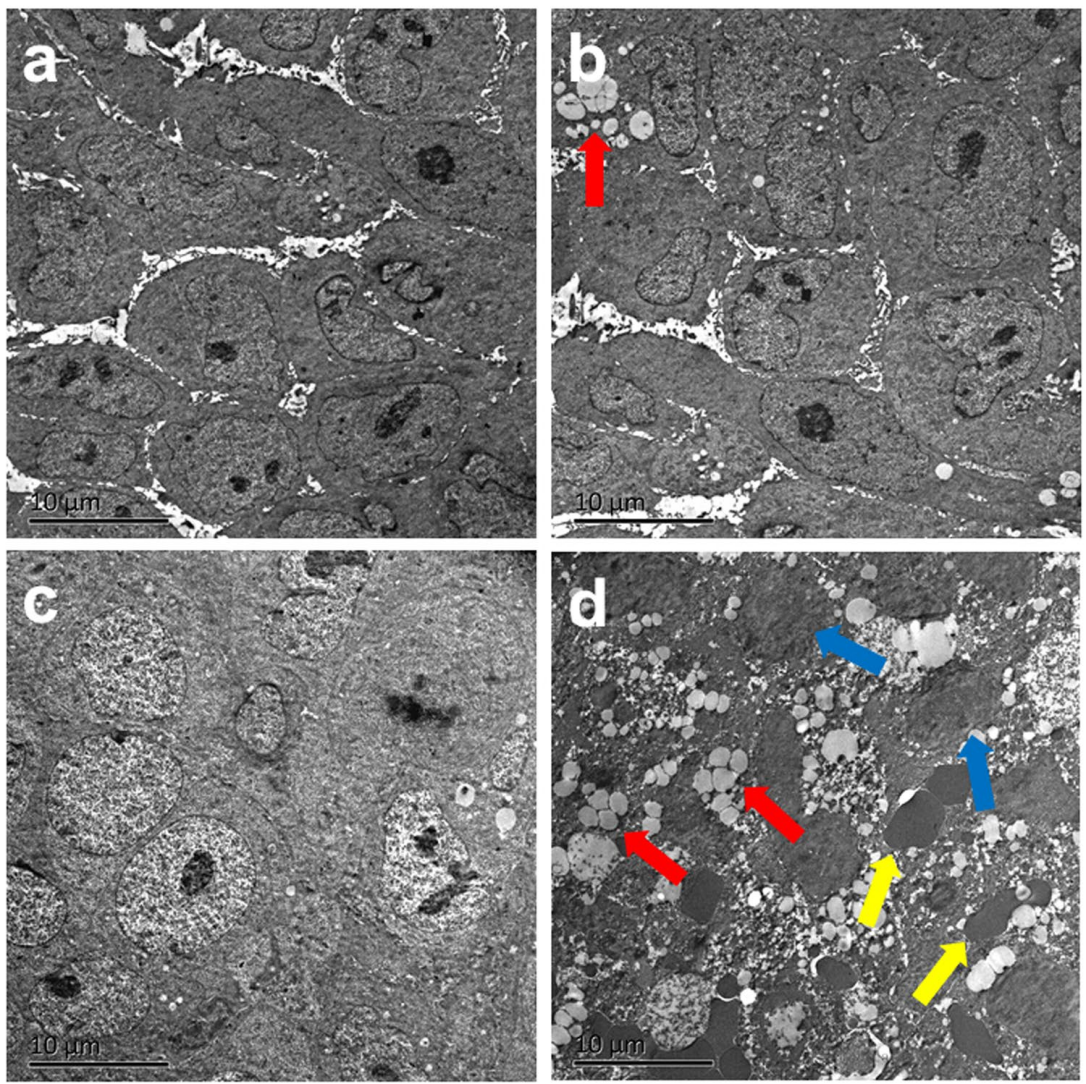
TEM images of tumor tissues from different treatment groups. (**a**) Saline; (**b**) Fe_3_O_4_/ICG@PLGA/PFP NPs only; (**c**) laser irradiation only; and (**d**) Fe_3_O_4_/ICG@PLGA/PFP NPs with laser irradiation. Magnification: 1450×; scale bar, 10 μm. Many generated microbubbles (red arrows) appeared in the tumor tissues. Blue arrows indicate interrupted cells, and yellow arrows indicate red blood cells.

The therapeutic effectiveness of Fe_3_O_4_/ICG@PLGA/PFP NPs in PTT was also investigated by H&E staining, TUNEL staining, and TEM examination of tumor tissues harvested from the different groups of mice 1 day after treatment. H&E staining revealed severe damage with coagulative necrosis, leaving a mass of red-stained substance in tumor tissue injected with NPs and exposed to laser irradiation. Comparatively, no notable damage was found in the three control groups (Fig. 6d). TUNEL staining further verified the presence of many more necrotic cells in tumor tissues of mice treated with the NPs and laser irradiation than in tumor tissues of the other three groups, demonstrating the greater damage to tumor cells achieved via the photothermal effect of Fe_3_O_4_/ICG@PLGA/PFP NPs (Fig. 6d). In addition, on TEM images, coagulative necrosis was more evident in the tumor tissues of mice treated with the NPs and laser irradiation than in that of the other three groups, which was consistent with our H&E staining results (Fig. 7). In the representative image showed in Fig. 7d, after treatment with NPs and laser irradiation, no clear cell structure could be distinguished, most cell membranes and nuclear membranes had ruptured or disintegrated (indicated by blue arrows), organelles had disappeared, and only remnants of the nuclear shadow remained. Moreover, the tumor blood vessels had ruptured, resulting in leakage of red blood cells. Comparatively, in the three control groups, the tumor tissue retained the original structure with no obvious damage (Fig. 7a–c). Finally, many microbubbles (red arrows) appeared in the tumor tissues treated with NPs and laser irradiation, with Fe particles expelled to the edge or outside of the microbubbles (Fig. 7d). This revealed that most Fe_3_O_4_/ICG@PLGA/PFP NPs absorbed the light energy and converted it into heat upon laser irradiation, and the PFP in the NPs underwent a phase shift to form microbubbles. In tumor tissues treated with NPs only without laser irradiation, only a small portion of NPs produced microbubbles (Fig. 7b). We think this may have occurred after harvesting of the tissue during the heated polymerization process of the TEM sample operation, when a temperature exceeding 70 °C is commonly used; at such temperatures, some of the NPs in the excised tissues could absorb the heat, resulting in microbubble formation.

### *In vivo* toxicity of Fe3O4/ICG@PLGA/PFP NPs

**Figure 8:**
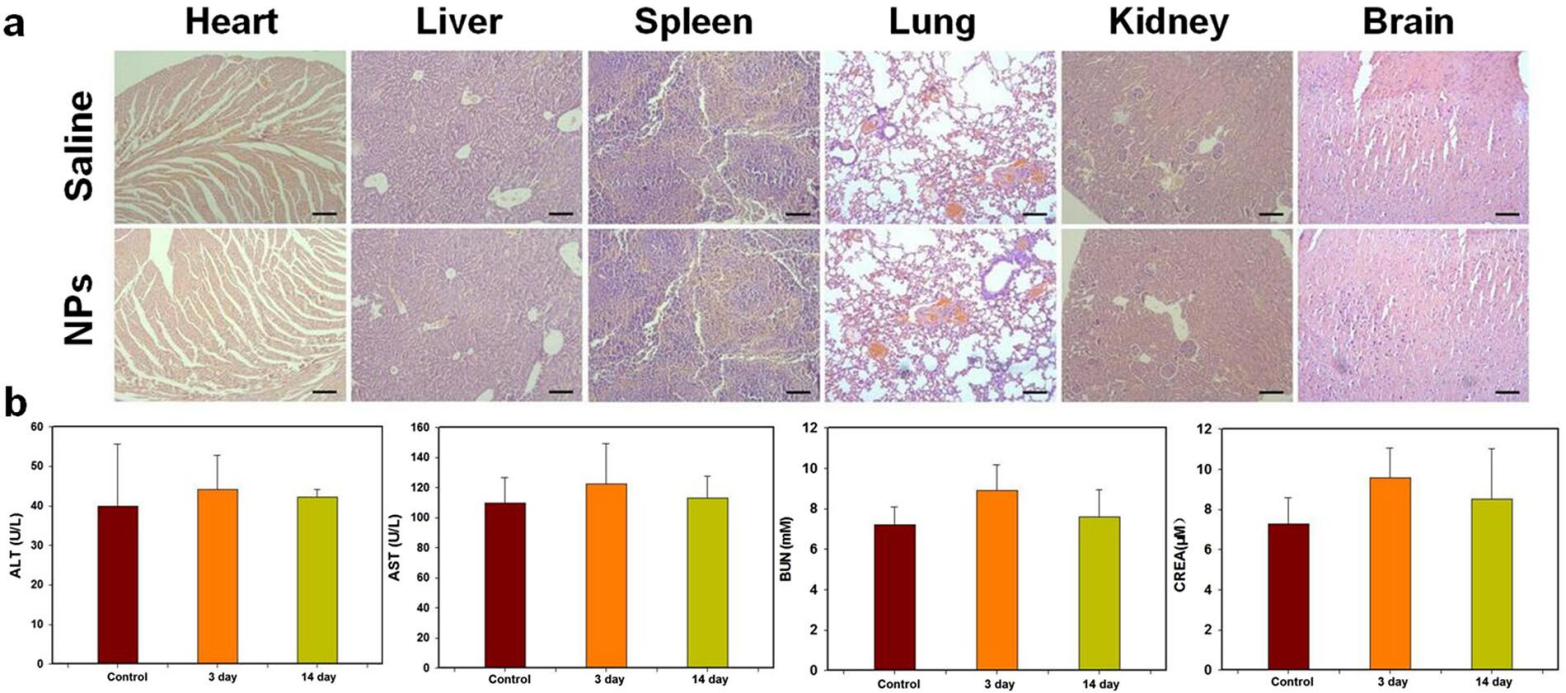
*In vivo* toxicity study. (**a**) H&E stained sections of major organs harvested 14 days after intravenous injection of 0.2 mL of 2 mg/mL Fe_3_O_4_/ICG@PLGA/PFP NPs or saline as the control. No noticeable abnormality was observed in the heart, liver, spleen, lung, kidney, or brain. (**b**) Levels of hepatic functional markers (ALT and AST) and renal functional markers (BUN and CREA) in the blood at 3 and 14 days after injection of Fe_3_O_4_/ICG@PLGA/PFP NPs or saline as the control.

To further assess the *in vivo* toxicity of Fe_3_O_4_/ICG@PLGA/PFP NPs, major organs (heart, liver, spleen, lung, kidney, and brain) of healthy BABL/c mice injected with Fe_3_O_4_/ICG@PLGA/PFP NPs (0.2 mL, 2 mg/mL) via the tail vein were harvested,sectioned, and stained with H&E for histological analysis at 14 days post-injection. In addition, blood samples of these healthy BABL/c mice were collected and analyzed at 3 and 14 days post-injection. No noticeable organ damage or inflammatory lesion were observed in H&E stained sections of major organs from mice treated with Fe_3_O_4_/ICG@PLGA/PFP NPs, indicating that the Fe_3_O_4_/ICG@PLGA/PFP NPs did not induce appreciable toxic side effects in treated animals (Fig. 8a). Moreover, two indicators of hepatic function, alanine aminotransferase (ALT) and aspartate aminotransferase (AST) levels, and two indicators of renal function, urea nitrogen (UREA) and creatinine (CREA) levels, were measured in blood samples from NP-treated mice. As shown in Fig. 8b, the levels of these four indicators were within normal ranges with no significant differences between the different time points (all *p < 0.05). Thus, the histological examination and serum biochemistry results indicate that Fe_3_O_4_/ICG@PLGA/PFP NPs at the tested dose have no cytotoxic effects in healthy mice in the absence of laser irradiation.

## Conclusion

The multifunctional Fe_3_O_4_/ICG@PLGA/PFP NPs synthesized in this study showed promising results as a photothermal agent for cancer treatment. The photo-absorbance by Fe_3_O_4_ NPs and ICG co-embedded in the Fe_3_O_4_/ICG@PLGA/PFP NP shell, along with the microbubbles generated upon the liquid-gas phase shift of PFP encapsulated in the NPs process make it possible to achieve photothermal tumor therapy. This successful demonstration of the use of nanobiotechnology for NIR laser-induced PTT provides an alternative modality for effective nanotheranostics.
